# Lameness*Abstract of Lecture recently delivered at Columbia Veterinary College.

**Published:** 1883-07

**Authors:** E. A. McLellan

**Affiliations:** Lecturer on Shoeing, Diseases of the Feet and Legs, etc., Columbia Veterinary College


### Art. XXIII.—LAMENESS.*

BY E. A. McLELLAN, D. V. S.

*Lecturer on Shoeing, Diseases of the Feet and Legs, etc., Columbia Veterinary College.*

LAMENESS in horses is a common condition. In our practice as veterinary surgeons we shall be called on daily for our diagnosis in cases of lameness, and for this reason we must avail ourselves of every means in our power to gain that knowledge which will make us expert in the Etiology, Diagnosis and Prognosis of lameness. Men without the special training which the college affords who have been accustomed to horses from their youth, become, many of them very proficient in locating lameness. These men you will meet, and your knowledge must exceed theirs, if you would maintain your standing.

The most important aid to proficiency is a habit of close observation. In this lecture I shall endeavor to point out the particular parts of the external form of the horse which you must accustom yourselves to observe. Among these we will place first as most important and the one requiring the most careful observation, the hoof. The feet of horses assume a great variety of forms. As we stated in a previous lecture the form of the hoof is the effect of two causes, namely—inheritance and environment, including under the latter term, the effects

*Abstract of Lecture recently delivered at Columbia Veterinary College.

of shoeing, stabling, etc., not including the effect of disease. To some of these forms of feet names have been applied, as, flat feet, those in which the sole presents a flat surface, instead of a concave one. Low-heeled feet, those in which the coronary circle touches the ground at the posterior part of the foot. Then we have large and small, wide and narrow, which terms are sufficiently descriptive.

Now, these differences in form are such as require but little experience and observation to distinguish, but there are others which are more difficult to acquire, such as the difference in the length of the foot posterior to the pedal bone; an abnormal condition of the plantar cushion; a position of the pedal bone in which its articular surface receives unequal pressure from the Os coronæ, and column of bones above; a diseased condition of the pyramidal eminence of pedal bone; a condition of the hoof which predisposes to fissures of the wall; unequal pressure at the coronet; the relation of the plantar surface to the coronary border of the wall, the relation of the foot to the leg; its position in relation to the fetlock; the manner in which the feet are placed upon the ground during progression and their relation to the body while at rest. These points, and many others, are important for you to observe, for the reason that the external form and appearance, give us a clue to the condition of the parts within, and also enable us to foretell what is likely to occur in the future. Their importance is still further emphasized by the fact, that of all horses lame in the anterior extremities, the cause of the lameness in 75 p. c. of the cases will be located beneath the hoof.

At this point we might consider the question as to whether the hoof does, under any circumstances injuriously press upon or bind, or constringe the sensitive foot. If you look through the literature of Veterniary Surgery you will discovery differing opinions upon this subject of contraction of the hoof; one set of writers holding that the hoof does contract and thereby produces disease and lameness; the other, that contraction is always the effect of disease. Among the latter, is Prof. Williams, who, in his Surgery, page 314, says: "Contraction of the hoof is not a cause but an effect of disease, an atrophy of the structures contained within the horny box consequent upon diminished functional activity and adaptability of the hoof to the atrophied structures, which it encloses and protects."

Another writer, Prof. Copeman, who at one time was teacher of Theory and Practice at the New York College of Veterinary Surgeons, takes about the same ground. He says: "Contraction of the hoof is more an imaginary than a real state. There is no alternate expansion and contraction going on in the foot, as has been supposed, during ordinary progression, neither is there any antagonism kept up by the hoof tending to constringe the vascular structures, and these in turn resisting that tendency, but complete harmony of parts, and counterparts, both in their form and action subsists between the hoof and all it encircles, the same as between the outer covering and subjacent structures of every animal in all its parts." These ideas are not in harmony with my own, and I am inclined to believe that the practice of these writers was not always consistent with their theory. Prof. Williams upon the same page from which I have quoted makes an admission which overthrows his theory. He says, in effect, that the hoof does become contracted as a result of injudicious pareing. But this admission is fatal to his position, for opening the heels is not "diminished functional activity." Prof. Copeman in his position as veterinary editor of *Spirit of the Times*, frequently gave direction to soak and poultice the feet which would seem quite uncalled for if the hoof did not, under certain circumstances, become dry and rigid, and thus "constringe the vascular structures."

My own view of the subject of contraction is, that owing to a variety of causes which we will not here consider in detail but which are included under the terms, shoeing, stabling, domestication, etc., that the relative position of the hoof becomes changed. That by this change a lack of harmony exists between the hoof and what it covers—that the vascular structures are constringed and suffering and disease result. Although I am perfectly satisfied that what I have stated is a fact, yet it is not an easy task to make this clear.

In the quotation which I made from Prof. Copeman, the relations which exist between the hoof and what it encloses, were compared to the relation which exists between the skin and subcutaneous tissues. Now, while this analogy is correct in so far as that the tissues are alike in their constituents elements, the hoof being a mass of epithleium, and also in part as to function, the hoof like the skin being protective, etc. ; yet, in one important respect in reference to this question of contraction, they are entirely dissimilar ; namely, in their *density* and *rigidity*. While the skin is soft and pliable, arranging itself in folds, or stretched to its utmost limit as the condition of the parts beneath demand, having in itself no power to mould the form, the hoof, hard and resisting, varying in thickness from one quarter to three quarters of an inch is not so adaptable, and has power to mould the soft structures within. The possibility of the harmony existing between the hoof and subjacent parts being disturbed, is rendered strong when we consider that its relation to the parts it covers may be changed suddenly by paring the plantar surface of the wall, or the application of a shoe. To explain; suppose, that viewed in profile the anterior wall and pastern, present the same angle, if we cut down the heels one-half an inch, we change the relation of the pedal bone to the Os coronæ, and, consequently, the direction in which the weight is thrown upon it. This change of position in the relation of the bones is opposed by the action of the ligaments and flexor tendons, their function being to restore if possible the parts to their normal relations. This they accomplish in part, but at the expense of the harmony between hoof and sensitive structures. If you visit the shoeing shop, and notice the plantar surface as it is pared, you will discover in many feet indications of this lack of harmony in the patches of discolored horn. These may appear at any point, but are most frequent close to the wall at the toe, about the point of the frog and at the heels—at the latter place receiving the popular name of corns.

Again, if the wall is permitted to grow deeper upon one side than the other a condition in which the hoof will constringe the vascular structures will be produced, and in this way, a certain depth of horn is demanded on each side to place the pedal bone enclosed, in the proper relation to the column of bones above forming the limb ; but if one side becomes deeper than the other, greater weight will be sustained by that side of the bone through its articular surface, and the greater the growth, the greater will be the disturbance of the pedal bone ; it will be pushed downwards bruising the sensitive tissue between bone and plantar horn. This anomaly is often noticed in the feet of cart horses, and in my experience produces the gravest results when found upon the outside quarter of the foot. The diagnosis of such a condition is obtained by a comparison of the depth of the horn upon each side, by the uneven wear of the shoe, by hypersensitiveness evinced when the the foot is hammered upon the affected side, and upon being pared a morbid condition of the plantar horn in the form of a red spot will be disclosed beneath the bar. The foot will also be rested or pointed with more or less lameness.

Here we have a case in which owing to a change in the part of the hoof which is presented to the ground, we have a constringing of the vascular and sensitive structures of the foot. Not necessarily in this case contraction of the hoof, but a change in the normal relations of the parts, which produces a lack of harmony between the hoof and sensitive parts within. That this is the abnormal condition of the parts which in such a case exists, is easily demonstrated. Let the wall be reduced upon the high side, let the plantar horn be thinned for a considerable distance around the part which indicates injurious pressure, that is about the red spot ; let a shoe be applied so as to relieve that quarter of the foot from pressure as much as possible, and improvement in the gait of the animal will be immediate, and entire removal of the lameness will speedily follow. Again, let me explain another of the factors which produces contraction of the hoof. The ordinary foot is so formed that pressure by the shoe upon the lower border of the wall posterior to the quarters, or widest part of the hoof, has a tendency to narrow the heels, that is, the pressure is not exerted in a vertical line parallel to the direction of the fibres of the wall, but there is a *lateral* or *inward* pressure as well. In the natural unshod foot the frog assists the wall, at the heels, in the weight bearing function, but when elevated from the ground by the shoe *its* accustomed support must be added to the pressure exerted upon the wall, and it is not unreasonable to believe that this lateral pressure, or pressure inwards and forwards which varies in degree according to the form of the heels, causes atrophy of the soft structures of the heels, and ultimately produced lameness by interference with the normal movements of the pedal bone or its appendages.

In that local inflammation in the posterior part of the foot known as corns, we have proof of injurious pressure. They may be either the result of maladjustment of hoof or shoe causing a change in the bearing of the pedal bone, or the result of this pressure upon the wall by the shoe. They are never, as by some supposed, the result of direct contact with the shoe, but the result of presure exerted upon the sensitive parts, through the medium of the hoof. In confirmation of this theory is the practice of almost every horse-shoer when the heels are much turned in, namely, to leave a space between shoe and wall at this point, to apply the shoe "easy on the heels" as it is said, to prevent lameness. This abnormal pressure by the shoe upon the quarters causes more or less pain and atrophy according to the form of the hoof at this point. If the heel be long and the soft parts of the foot largely developed this atrophy may go on for a long time without materially affecting the gait, but if the heel be short, lameness quickly results. The lameness may be hastened by permitting the hoof to become overgrown or rigid from a long period of rest in the stable, as a result of sickness, especially if there has been much elevation in the temperature of the body. There are many other causes which might be mentioned as producing a condition in which the hoof is caused to constringe, or bind the vascular and sensitive structures within; but the strongest proof that such is the case is found in the fact that a removal of the contraction gives relief from lameness, and this proof I have thoroughly tested. The various methods which can be employed with safety to accomplish this result will be explained at some future time.

To recapitulate, we believe that contraction of the hoof is a cause of pain and disease. 1st.—That owing to the density of the hoof and its modification by growth, it has *power* to injure the sensitive parts enclosed. 2d.—That owing to a peculiarity in the form of the heel of the ordinary foot, and the unnatural process of shoeing, injurious pressure is exerted upon the posterior parts of the foot; and 3d.—That contraction is proved as a cause of lameness and disease by the fact that remedies which relieve the contraction relieve the lameness.

I have laid stress upon this subject of contraction, because I consider it important, that it is one of the fundemental facts which must be remembered in our discussion of our foot troubles. The history of Veterniary Surgery shows that its students have been divided upon this subject, one party going to the extreme in belief that contraction of the hoof was the cause of all foot and leg ailments, the other going to the other extreme, considering contraction always an effect of lameness and disease in the foot or limb. I believe their is a middle ground which is tenable between these opposing beliefs. Pressure by the hoof is a cause of lameness and disease, but of not *all* lameness. Contraction of the hoof is an effect of lameness, but is often a cause. A practical question just here is, how shall we know from external appearances whether contraction exists or not in a foot? There may not be lameness, the impairment of the action may be scarcely noticeable, and yet a pressure by the hoof and wasting has taken place. This we can determine by *inspection.* In the foot suffering from contraction the face of the wall posterior to the widest part of the foot is *concave* with the concavity looking outwards. Again, in the normal foot, the cornary circle, or superior border of the wall, should present nearly a straight line from the toe to heel, but in the contracted foot, from the quarters backward the line will be curved upwards and then downwards; the horizontal lines in the wall, also will be noticed to follow this same direction.

We observe then the form of the hoof, and become familiar with that form which is likely to be associated with soundness, and also with lameness.

We next observe the *condition* of the hoof. Is it smooth, indicating a normal secretion, or rough, scaly, with fissues, or ridges? Is the frog sound and well formed, or is it ragged or shrunken with a discharge from its medium lacunæ?

One point that I wish to direct your attention to, is the condition of the plantar cushion, as indicated at its posterior portion. Is it thickened? In the normal foot, the hollow in the heels leaves the heel bulbs well defined, but in navicular arthritis, this thickening is usually well defined. Some thickening can usually be detected, even in the incipient stage of this formidable disease, so that this sign becomes valuable in diagnosis. Another point necessary to examine carefully is the condition of the lateral cartilages of the pedal bone. Are they flexable or hard? If bone is occupying the place of these cartilages to any extent the elasticity of the step will be impaired, and if the animal is used for the carriage a long, hard drive is almost sure to produce lameness.

Another point which may assist diagnosis is to observe the relation of the foot to the leg.

In some cases you will notice that the toe turns in, and the heels are turned out, giving the appearance of having been pivoted in this direction on its articular surface. In this case the pastern stands directly over the inside heel. Such a condition predisposes to injurious pressure upon the inside quarter of the foot to corns and fissures of the wall.

In another horse we find a long pastern. The foot is placed upon the ground far in advance of the leg. In such a case great strain is put upon the ligaments which bind the articulations, and upon the flexors. In this form of leg a common seat for a lesion is the fetlock, either spavin of the suspensory ligament, the lateral sessamoid ligaments, or a sessamoidities. If the toe of the hoof is short, and its anterior face approaches the vertical, while the pastern being long it varied toward the horizontal, we might look for the lesion in the anterior lateral ligaments of the pedal articulation, while in case the toe of the hoof be long, and in profile presenting the same angle as the pastern, injurious pressure at the coronet by the hoof in front might be suspected. The first is more common in the hind feet, the last in the front feet. In the case of a long pastern, allowing the hoof to become excessively grown, greatly increases the danger of sprain of some portion of the leg.

Again, the manner in which the shoe is worn furnishes an aid to diagnosis. If worn excessively upon one side, as has been already stated, we suspect maladjustment of the hoof. If the wear is greatest at the toe we have a form of the limb which places extreme tension upon the flexors; the wear being caused by the toe impinging upon the ground at the beginning of the act of flexion, or by a lack of motion in the pedal articulation, the foot being obliged to pivot upon the toe as the body is carried forward, as is the case in navicular disease, or ossified cartilages, or any condition in which the elasticity of these parts is impaired. If the wear is greatest at the heel, we look for the lesion at the anterior portion of the foot, as from lamminitis or fissures in the wall at the toe.

We next observe the the position of feet and legs in their relation to the body. The horse when standing quietly assumes that position which brings the greatest repose. We must become familiar with the normal relative position of all the parts to detect any change from this, and also be able from our knowledge of the anatomy, and our general experience to determine the seat of the lesion from the position assumed. For example, when the hocks are the seat of the disease, the fore limbs are kept well back under the body, and the anterior border of the sternum is prominent, on the other hand, when the fore feet are diseased as in Laminitis, the hind feet are carried forward, the scapulo-humeral articulation is prominent and the sternum is retracted. This much in a general way, but in the discussion of the different diseased conditions of the feet and legs at some future time these points will be fully noticed. In passing up the leg we come next to the knee. Here we may have an enlarged condition, a distention of bursæ, or nodular growths. Enlargements on its inner aspect suggest interfering, which, perhaps, is not unsoundness, but a serious defect, which frequently ends in an unsound condition. Exostoses on the limb must be noticed if present. They are particularly grave, when situated on the pastern. We notice the condition of the shoulders. What is the appearance of the muscular covering? Is the spine of the scapula prominent, with a thin covering of muscle above it? Associated with this is the point of the shoulder prominent, giving hollowness to the chest in front. If so, you have indications of disease of the feet. Always view with suspicion the horse, even if he is going apparently sound, if the muscles are wasted, particularly at the superior portion of the shoulder.

In the hind limbs, the region of the hock, demands our closest attention. It must be viewed at every angle. When its confirmation leads us to suspect disease, we must closely inspect, and in motion carefully note the manner in which the first few steps are taken. An inspection of the shoe is of value. In hock-lameness the toe is always most worn. Holding the foot in the position in which the shoer nails on the shoe will usually cause the animal pain, occuring in exostosis on the anterior border of the joint. A difference in temperature, if one only is diseased, can frequently be detected by applying a hand to each hock. In all the lesions of the feet and limbs, which we have mentioned, we must supplement our occular examinations with palpation, manipulations by the hand to discover pain, heat, etc. Lesions in the region of the patella are usually referable to accidents, and their presence best detected from motion.

Fractures of the anterio-external angle of the ilium are frequent and best detected by standing behind the animal. Having looked our horse over carefully while at rest for defects in his locomotary apparatus, we now proceed to inspect him while in motion.

*(To be continued.)*

